# Novel 2,4,6-Trimethylbenzenesulfonyl Hydrazones with Antibacterial Activity: Synthesis and In Vitro Study

**DOI:** 10.3390/ma14112723

**Published:** 2021-05-21

**Authors:** Łukasz Popiołek, Sylwia Szeremeta, Anna Biernasiuk, Monika Wujec

**Affiliations:** 1 Department of Organic Chemistry, Faculty of Pharmacy, Medical University of Lublin, 4A Chodźki Street, 20-093 Lublin, Poland; sszeremeta96@o2.pl (S.S.); monika.wujec@umlub.pl (M.W.); 2 Department of Pharmaceutical Microbiology, Faculty of Pharmacy, Medical University of Lublin, 1 Chodźki Street, 20-093 Lublin, Poland; anna.biernasiuk@umlub.pl

**Keywords:** benzenesulfonyl hydrazones, bioactivity, antibacterial activity, MIC, MBC, ligands

## Abstract

This research describes the synthesis and in vitro antimicrobial activity study of a series of 2,4,6-trimethylbenzenesulfonyl hydrazones. Twenty-five hydrazones (**2**–**26**) were synthesized on the basis of condensation reaction. The in vitro bioactivity study confirmed the potential application of obtained derivatives as antimicrobial agents. Among the tested compounds, the highest activity was discovered for derivative 24, which possessed minimal inhibitory concentration (MIC) ranging from 7.81 to 15.62 µg/mL against Gram-positive reference bacterial strains. Synthesized benzenesulfonyl hydrazones can be applied as potential ligands for the synthesis of bioactive metal complexes.

## 1. Introduction

In recent years, the problem of the emerging resistance of bacteria and fungi to antibiotics and chemotherapeutic agents has been observed [1]. Even 30–40 years ago, this problem was not so noticeable because from the 1940s to the 1980s, the pharmaceutical industry introduced many classes of medicines to the treatment [1,2,3]. In the fight against bacterial and fungal infections, antimicrobial agents are used, which can be divided into two main classes: obtained by isolating from naturally occurring organisms in the ecosystem and by chemical modification of them, namely, antibiotics, and obtained by chemical synthesis, namely, chemotherapeutic agents. Recent years have shown that antimicrobial agents that have been used for decades are not always effective in treating infections [1,2,3]. Due to these factors, scientists are seeking new molecules with antimicrobial properties. The discovery of new medicines with antimicrobial activity profile could eliminate infections that the current healthcare system cannot cope with, and it would also be possible to shorten hospitalization time and possible complications, as well as to reduce the number of therapeutic agents used [1,2,3].

One class of compounds that has attracted the attention of scientists in recent years is benzenesulfonohydrazide derivatives. This interest results from the possibility of their wide use in chemical synthesis as intermediates [4,5,6,7], as well as the fact that this group of compounds has significant and wide spectrum of biological properties, including anticancer [8,9,10,11,12], antibacterial [13,14,15,16,17], antifungal [14,18,19,20,21], and antidepressant activity [22,23].

**Figure 1:**
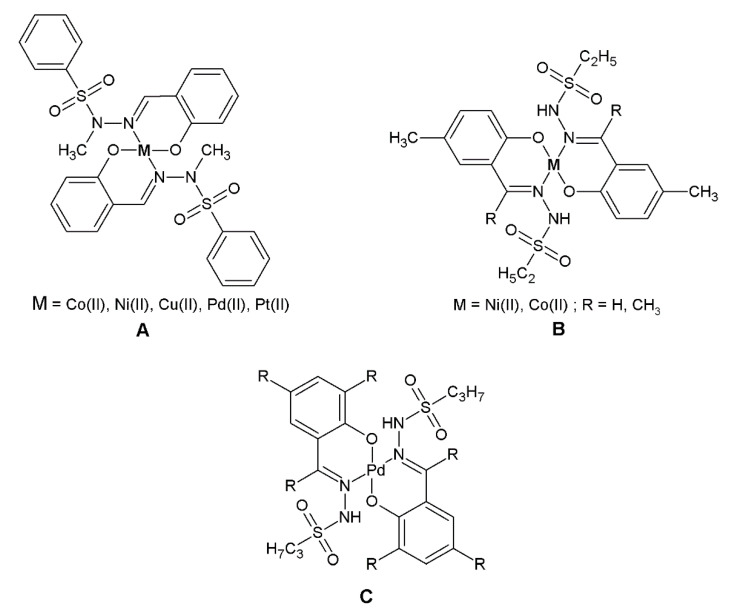
Benzenesulfonyl hydrazone complexes with antibacterial activity (**A**–**C**).

Benzenesulfonyl hydrazones also play an important role in coordination chemistry as ligands in the synthesis of metal complexes [24,25,26,27,28,29,30,31]. This is due to the fact that they possess a number of possible metal binging sites and can act as both bi- and tetradentate ligands, depending on the portion of the ligand involved in the metal complexation [28]. According to scientific reports, such complexes display interesting biological activity. They possess mainly antibacterial [24,25,26,27,28], anticancer [29], and antimalarial properties [30]. Aslan et al. reported the synthesis of novel benzenesulfonic acid derivatives and its Ni(II), Pd(II), Pt(II), Cu(II), and Co(II) complexes (Figure 1A) [24]. Synthesized compounds showed potent inhibition against tested bacterial strains [24]. Similar complexes with significant antibacterial activity were also synthesized by Özdemir et al. (Figure 1B) [25] and Özbek et al. (Figure 1C) [27].

In the scientific literature findings, there is also some information that concerns the synthesis and activity of organometallic-based sulfonyl hydrazones [31]. Concha et al. synthesized series of organometallic tosylhydrazones containing cyrhetrenyl and cymantrenyl moieties and evaluated them for potential antitubercular and antitumor activity [31].

Prompted by the above-mentioned fact, in this research, we designed, synthesized, and tested for in vitro antimicrobial activity a novel series of 2,4,6-trimethylbenzenesulfonyl hydrazones with the aim to obtain promising potential antimicrobial agents that can be used further as potential ligands for the synthesis of bioactive metal complexes.

## 2. Materials and Methods

### 2.1. Chemistry

All reagents and solvents used in this research were purchased from Sigma-Aldrich (Munich, Germany) and Merck Co. (Darmstadt, Germany) and used without further purification. Thin-layer chromatography (TLC) on plates covered with silica gel (aluminum oxide 60 F-254, Merck Co., Kenilworth, NJ, USA) was used to check the purity of the obtained compounds and to monitor the progress of the reaction. Chloroform–ethanol mixture in the 10:1 (*v*/*v*) ratio was used as the mobile phase. The spots were detected by irradiation with UV light at a wavelength of λ = 254 nm. ^1^H NMR and ^13^C NMR spectra were recorded on the Bruker Avance 300 and 600 apparatus (Bruker BioSpin GmbH, Rheinstetten, Germany). The melting points of the obtained compounds were measured with a Fisher–Johns apparatus (Fisher Scientific, Schwerte, Germany) and presented without any correction. The elemental analysis was determined by the Perkin Elmer 2400 series II CHNS/O analyzer (Waltham, MA, USA), and the results were within ±0.4% of the theoretical value.

#### 2.1.1. Preparation of 2,4,6-Trimethylbenzenesulfonyl Hydrazones (**2**–**26**)

The 2,4,6-trimethylbenzenesulfonyl hydrazones were synthesized with the use of the method described earlier by our group for the synthesis of hydrazide–hydrazones [32,33,34].

**Scheme 1:**
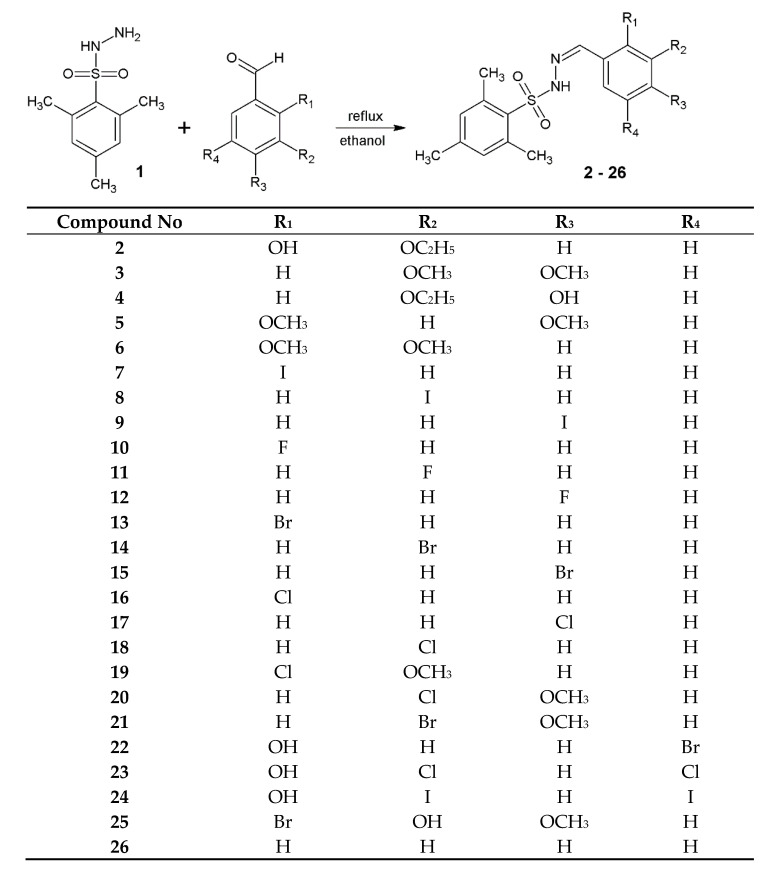
The synthesis of novel 2,4,6-trimethylbenzenesulfonyl hydrazones.

We dissolved 0.01 mole of 2,4,6-trimethylbenzenesulfonohydrazide (**1**) in ethanol (5 mL, 96%). Then, 0.011 mole of appropriate substituted benzaldehyde was added, and the mixture was heated under reflux for 3 h. The substituted benzaldehydes used in this research are presented in Scheme 1. After that, the solution was cooled to room temperature and placed in the refrigerator for 24 h. Subsequently the precipitate formed was filtered off and re-crystallized from ethanol (96%).

#### 2.1.2. Physicochemical Properties of 2,4,6-Trimethylbenzenesulfonyl Hydrazones (**2**–**26**)

*N*-[(3-Ethoxy-2-hydroxyphenyl)methylidene]-2,4,6-trimethylbenzenesulfonohydrazide (**2**)

Yellow powder; CAS Number: 1799087-18-5; M.p.: 132 °C; Yield: 52%; ^1^H NMR (300 MHz, DMSO-*d*_6_) δ (ppm): 1.29–1.31 (t, 3H, CH_3_, *J* = 9 Hz, *J* = 6 Hz), 2.24 (s, 3H, CH_3_), 2.62 (s, 6H, 2 × CH_3_), 3.98–4.05 (q, 2H, CH_2_, *J* = 6 Hz), 6.71–6.76 (t, 1H, ArH, *J* = 9 Hz, *J* = 6 Hz), 6.86–6.92 (m, 1H, ArH), 6.94–7.00 (m, 1H, ArH), 7.05 (s, 2H, ArH), 8.20 (s, 1H, =CH), 8.98 (s, 1H, OH), 11.54 (s, 1H, NH); ^13^C NMR (75 MHz, DMSO-*d*_6_) δ (ppm): 15.18 (CH_3_), 21.63 (2 × CH_3_), 23.14 (CH_3_), 64.52 (CH_2_), 118.85, 120.19, 132.16, 133.49, 139.64, 142.86, 146.53, 147.45 (10 × C_ar_), 147.54 (=CH), 149.22, 163.38 (2 × C_ar_).

*N*-[(3,4-Dimethoxyphenyl)methylidene]-2,4,6-trimethylbenzenesulfonohydrazide (**3**)

Yellowish powder; CAS Number: 1799143-26-2; M.p.: 140 °C; Yield: 61%; ^1^H NMR (300 MHz, DMSO-*d*_6_) δ (ppm): 2.23 (s, 3H, CH_3_), 2.65 (s, 6H, 2 × CH_3_), 3.72 (s, 3H, OCH_3_), 3.75 (s, 3H, OCH_3_), 6.93–6.95 (d, 1H, ArH, *J* = 6 Hz), 7.04 (s, 2H, ArH), 7.05–7.08 (m, 1H, ArH), 7.81 (s, 1H, ArH), 8.64 (s, 1H, =CH), 11.38 (s, 1H, NH); ^13^C NMR (75 MHz, DMSO-*d*_6_) δ (ppm): 20.87 (2 × CH_3_), 23.23 (CH_3_), 56.79 (OCH_3_), 57.89 (OCH_3_), 111.91, 114.10, 122.11, 127.07, 129.35, 132.05, 133.84, 139.73 (10 × C_ar_), 142.68 (=CH), 149.32, 150.88 (2 × C_ar_).

*N*-[(3-Ethoxy-4-hydroxyphenyl)methylidene]-2,4,6-trimethylbenzenesulfonohydrazide (**4**)

Brown powder; CAS Number: 1799022-47-1; M.p.: 142 °C; Yield: 52%; ^1^H NMR (300 MHz, DMSO-*d*_6_) δ (ppm): 1.30–1.32 (t, 3H, CH_3_), 2.23 (s, 3H, CH_3_), 2.64 (s, 6H, 2 × CH_3_), 3.94–4.01 (q, 2H, CH_2_, *J* = 9 Hz, *J* = 6 Hz), 6.74–6.77 (d, 1H, ArH, *J* = 9 Hz), 6.86–6.90 (m, 1H, ArH), 7.03 (s, 2H, ArH), 7.22–7.25 (d, 1H, ArH, *J* = 9 Hz), 7.76 (s, 1H, OH), 8.55 (s, 1H, =CH), 11.26 (s, 1H, NH); ^13^C NMR (75 MHz, DMSO-*d*_6_) δ (ppm): 15.10 (CH_3_), 20.87 (2 × CH_3_), 23.24 (CH_3_), 64.52 (CH_2_), 115.92, 125.74, 125.88, 132.03, 133.88, 139.71, 142.62, 146.26 (10 × C_ar_), 147.57 (=CH), 149.35, 150.57 (2 × C_ar_).

*N*-[(2,4-Dimethoxyphenyl)methylidene]-2,4,6-trimethylbenzenesulfonohydrazide (**5**)

Orange powder; CAS Number: 1799183-35-9; M.p.: 140 °C; Yield: 45%; ^1^H NMR (300 MHz, DMSO-*d*_6_) δ (ppm): 2.23 (s, 3H, CH_3_), 2.61 (s, 6H, 2 × CH_3_), 3.76 (s, 3H, OCH_3_), 3.78 (s, 3H, OCH_3_), 6.53–6.55 (m, 2H, ArH), 7.02 (s, 2H, ArH), 7.42–7.45 (d, 1H, ArH, *J* = 9 Hz), 8.13 (s, 1H, =CH), 11.23 (s, 1H, NH); ^13^C NMR (75 MHz, DMSO-*d*_6_) δ (ppm): 20.86 (2 × CH_3_), 23.21 (CH_3_), 56.04 (OCH_3_), 56.79 (OCH_3_), 98.66, 106.87, 115.09, 126.39, 132.04, 133.90, 139.63, 141.62 (10 × C_ar_), 142.58 (=CH), 159.17, 162.70 (2 × C_ar_).

*N*-[(2,3-Dimethoxyphenyl)methylidene]-2,4,6-trimethylbenzenesulfonohydrazide (**6**)

White powder; CAS Number: 1799021-56-9; M.p.: 118 °C; Yield: 43%; ^1^H NMR (300 MHz, DMSO-*d*_6_) δ (ppm): 2.23 (s, 3H, CH_3_), 2.63 (s, 6H, 2 × CH_3_), 3.70 (s, 3H, OCH_3_), 3.79 (s, 3H, OCH_3_), 7.03 (s, 2H, ArH), 7.04–7.12 (m, 3H, ArH), 8.17 (s, 1H, =CH), 11.54 (s, 1H, NH); ^13^C NMR (75 MHz, DMSO-*d*_6_) δ (ppm): 20.89 (2 × CH_3_), 23.20 (CH_3_), 56.81 (OCH_3_), 56.99 (OCH_3_), 112.11, 115.09, 123.23, 127.77, 129.15, 132.75, 133.64, 138.13 (10 × C_ar_), 142.78 (=CH), 150.31, 151.98 (2 × C_ar_).

*N*-[(2-Iodophenyl)methylidene]-2,4,6-trimethylbenzenesulfonohydrazide (**7**)

Bright yellow powder; M.p.: 170 °C; Yield: 76%; ^1^H NMR (300 MHz, DMSO-*d*_6_) δ (ppm): 2.24 (s, 3H, CH_3_), 2.63 (s, 6H, 2 × CH_3_), 7.05 (s, 2H, ArH), 7.08–7.14 (m, 1H, ArH), 7.36–7.41 (t, 1H, ArH, *J* = 6 Hz, *J* = 9 Hz), 7.53–7.56 (d, 1H, ArH, *J* = 9 Hz), 7.85–7.88 (d, 1H, ArH, *J* = 9 Hz), 8.13 (s, 1H, =CH), 11.87 (s, 1H, NH); ^13^C NMR (75 MHz, DMSO-*d*_6_) δ (ppm): 20.89 (CH_3_), 23.18 (2 × CH_3_), 100.15, 126.57, 129.07, 132.08, 132.17, 133.70, 135.67, 139.60, 140.15 (11 × C_ar_), 142.91 (=CH), 148.51 (C_ar_).

*N*-[(3-Iodophenyl)methylidene]-2,4,6-trimethylbenzenesulfonohydrazide (**8**)

Orange powder; M.p.: 128 °C; Yield: 78%; ^1^H NMR (300 MHz, DMSO-*d*_6_) δ (ppm): 2.24 (s, 3H, CH_3_), 2.63 (s, 6H, 2 × CH_3_), 7.05 (s, 2H, ArH), 7.15–7.20 (t, 1H, ArH, *J* = 9 Hz, *J* = 6 Hz), 7.49–7.51 (d, 1H, ArH, *J* = 6 Hz), 7.69–7.72 (m, 1H, ArH), 7.86–7.90 (m, 1H, ArH), 7.83 (s, 1H, =CH), 11.76 (s, 1H, NH); ^13^C NMR (75 MHz, DMSO-*d*_6_) δ (ppm): 20.89 (CH_3_), 23.16 (2 × CH_3_), 95.68, 126.21, 131.39, 132.14, 133.73, 135.26, 136.58, 138.66, 139.63 (11 × C_ar_), 142.88 (=CH), 143.88 (C_ar_).

*N*-[(4-Iodophenyl)methylidene]-2,4,6-trimethylbenzenesulfonohydrazide (**9**)

Light yellow powder; M.p.: 166 °C; Yield: 46%; ^1^H NMR (300 MHz, DMSO-*d*_6_) δ (ppm): 2.23 (s, 3H, CH_3_), 2.62 (s, 6H, 2 × CH_3_), 7.04 (s, 2H, ArH), 7.26–7.29 (d, 2H, ArH, *J* = 9 Hz), 7.88–7.91 (d, 2H, ArH, *J* = 9 Hz), 8.67 (s, 1H, =CH), 11.68 (s, 1H, NH); ^13^C NMR (75 MHz, DMSO-*d*_6_) δ (ppm): 21.03 (CH_3_), 23.19 (2 × CH_3_), 99.45, 128.75, 130.55, 132.12, 133.66, 138.12, 138.33, 139.64 (12 × C_ar_), 142.82 (=CH).

*N*-[(2-Fluorophenyl)methylidene]-2,4,6-trimethylbenzenesulfonohydrazide (**10**)

Bright yellow powder; CAS Number: 1799182-76-5; M.p.: 161 °C; Yield: 54%; ^1^H NMR (300 MHz, DMSO-*d*_6_) δ (ppm): 2.24 (s, 3H, CH_3_), 2.63 (s, 6H, 2 × CH_3_), 7.05 (s, 2H, ArH), 7.19–7.27 (m, 1H, ArH), 7.35–7.45 (m, 1H, ArH), 7.55–7.61 (m, 1H, ArH), 7.85–7.88 (d, 1H, ArH, *J* = 9 Hz), 8.09 (s, 1H, =CH), 11.77 (s, 1H, NH); ^13^C NMR (150 MHz, DMSO-*d*_6_) δ (ppm): 20.89 (CH_3_), 23.15 (2 × CH_3_), 116.61, 121.77, 125.41, 126.22, 132.14, 133.68, 138.53, 139.72 (10 × C_ar_), 142.89 (=CH), 155.57, 159.99 (2 × C_ar_).

*N*-[(3-Fluorophenyl)methylidene]-2,4,6-trimethylbenzenesulfonohydrazide (**11**)

Bright yellow powder; CAS Number: 1799086-95-5; M.p.: 126 °C; Yield: 7%; ^1^H NMR (300 MHz, DMSO-*d*_6_) δ (ppm): 2.26 (s, 3H, CH_3_), 2.62 (s, 6H, 2 × CH_3_), 7.07 (s, 2H, ArH), 7.20–7.28 (m, 1H, ArH), 7.38–7.44 (m, 1H, ArH), 7.59–7.65 (m, 1H, ArH), 7.88–7.90 (d, 1H, ArH, *J* = 9 Hz), 8.19 (s, 1H, =CH), 11.97 (s, 1H, NH); ^13^C NMR (150 MHz, DMSO-*d*_6_) δ (ppm): 20.90 (CH_3_), 23.20 (2 × CH_3_), 117.62, 121.87, 126.11, 126.72, 132.34, 133.98, 138.23, 139.92 (10 × C_ar_), 142.95 (=CH), 155.67, 159.19 (2 × C_ar_).

*N*-[(4-Fluorophenyl)methylidene]-2,4,6-trimethylbenzenesulfonohydrazide (**12**)

Orange powder; CAS Number: 1799193-67-1; M.p.: 168 °C; Yield: 40%; ^1^H NMR (300 MHz, DMSO-*d*_6_) δ (ppm): 2.23 (s, 3H, CH_3_), 2.63 (s, 6H, 2 × CH_3_), 7.04 (s, 2H, ArH), 7.19–7.25 (m, 2H, ArH), 7.52–7.57 (m, 2H, ArH), 7.90 (s, 1H, =CH), 11.60 (s, 1H, NH); ^13^C NMR (150 MHz, DMSO-*d*_6_) δ (ppm): 20.88 (CH_3_), 23.19 (2 × CH_3_), 116.29, 129.07, 132.10, 133.80, 139.67, 144.76 (10 × C_ar_), 142.76 (=CH), 160.89, 162.57 (2 × C_ar_).

*N*-[(2-Bromophenyl)methylidene]-2,4,6-trimethylbenzenesulfonohydrazide (**13**)

Yellow powder; M.p.: 156 °C; Yield: 14%; ^1^H NMR (300 MHz, DMSO-*d*_6_) δ (ppm): 2.24 (s, 3H, CH_3_), 2.63 (s, 6H, 2 × CH_3_), 7.05 (s, 2H, ArH), 7.27–7.32 (m, 1H, ArH), 7.39–7.41 (t, 1H, ArH, *J* = 3 Hz), 7.48–7.53 (m, 1H, ArH), 7.60–7.65 (m, 1H, ArH), 8.24 (s, 1H, =CH), 11.88 (s, 1H, NH); ^13^C NMR (150 MHz, DMSO-*d*_6_) δ (ppm): 20.90 (CH_3_), 23.16 (2 × CH_3_), 123.58, 126.85, 128.61, 129.06, 132.97, 133.68, 133.91, 139.62 (10 × C_ar_), 142.93 (=CH), 143.87, 161.24 (2 × C_ar_).

*N*-[(3-Bromophenyl)methylidene]-2,4,6-trimethylbenzenesulfonohydrazide (**14**)

Bright yellow powder; CAS Number: 1799080-75-3; M.p.: 150 °C; Yield: 73%; ^1^H NMR (300 MHz, DMSO-*d*_6_) δ (ppm): 2.23 (s, 3H, CH_3_), 2.63 (s, 6H, 2 × CH_3_), 7.05 (s, 2H, ArH), 7.31–7.36 (t, 1H, ArH, *J* = 6 Hz, *J* = 9 Hz), 7.48–7.57 (m, 2H, ArH), 7.67–7.68 (m, 1H, ArH), 7.87 (s, 1H, =CH), 11.79 (s, 1H, NH); ^13^C NMR (150 MHz, DMSO-*d*_6_) δ (ppm): 20.89 (CH_3_), 23.15 (2 × CH_3_), 122.55, 125.86, 129.35, 131.46, 132.14, 132.84, 133.75, 136.76, 139.69 (11 × C_ar_), 142.88 (=CH), 143.90 (C_ar_).

*N*-[(4-Bromophenyl)methylidene]-2,4,6-trimethylbenzenesulfonohydrazide (**15**)

Bright yellow powder; CAS Number: 1799088-79-1; M.p.: 155 °C; Yield: 67%; ^1^H NMR (300 MHz, DMSO-*d*_6_) δ (ppm): 2.24 (s, 3H, CH_3_), 2.62 (s, 6H, 2 × CH_3_), 7.04 (s, 2H, ArH), 7.42–7.45 (d, 2H, ArH, *J* = 9 Hz), 7.57–7.60 (d, 2H, ArH, *J* = 9 Hz), 7.88 (s, 1H, =CH), 11.70 (s, 1H, NH); ^13^C NMR (150 MHz, DMSO-*d*_6_) δ (ppm): 20.89 (CH_3_), 23.19 (2 × CH_3_), 123.55, 128.80, 132.12, 132.31, 133.57, 133.76, 139.66 (11 × C_ar_), 142.82 (=CH), 144.53 (C_ar_).

*N*-[(2-Chlorophenyl)methylidene]-2,4,6-trimethylbenzenesulfonohydrazide (**16**)

Yellow powder; CAS Number: 1799120-48-1; M.p.: 125 °C; Yield: 35%; ^1^H NMR (300 MHz, DMSO-*d*_6_) δ (ppm): 2.24 (s, 3H, CH_3_), 2.63 (s, 6H, 2 × CH_3_), 7.05 (s, 2H, ArH), 7.32–7.41 (m, 1H, ArH), 7.45–7.51 (m, 1H, ArH), 7.57–7.65 (m, 2H, ArH), 8.98 (s, 1H, =CH), 11.87 (s, 1H, NH); ^13^C NMR (75 MHz, DMSO-*d*_6_) δ (ppm): 20.89 (CH_3_), 23.17 (2 × CH_3_), 126.49, 128.68, 130.69, 131.41, 132.17, 133.18, 135.15, 139.64, 141.49 (11 × C_ar_), 142.95 (=CH), 158.77 (C_ar_).

*N*-[(4-Chlorophenyl)methylidene]-2,4,6-trimethylbenzenesulfonohydrazide (**17**)

Yellow powder; CAS Number: 1798924-70-5; M.p.: 122 °C; Yield: 29%; ^1^H NMR (300 MHz, DMSO-*d*_6_) δ (ppm): 2.24 (s, 3H, CH_3_), 2.63 (s, 6H, 2 × CH_3_), 7.04 (s, 2H, ArH), 7.43–7.46 (d, 1H, ArH, *J* = 9 Hz), 7.49–7.52 (d, 1H, ArH, *J* = 9 Hz), 7.57–7.60 (d, 1H, ArH, *J* = 9 Hz), 7.89–7.92 (d, 1H, ArH, *J* = 9 Hz), 8.72 (s, 1H, =CH), 11.70 (s, 1H, NH); ^13^C NMR (150 MHz, DMSO-*d*_6_) δ (ppm): 20.89 (CH_3_), 23.19 (2 × CH_3_), 128.57, 129.29, 130.51, 132.12, 133.09, 133.23, 133.77, 134.81, 136.56, 139.66 (10 × C_ar_), 142.82 (=CH), 144.44, 161.08 (2 × C_ar_).

*N*-[(3-Chlorophenyl)methylidene]-2,4,6-trimethylbenzenesulfonohydrazide (**18**)

Bright yellow powder; CAS Number: 1799187-65-7; M.p.: 140–143 °C; Yield: 41%; ^1^H NMR (600 MHz, DMSO-*d*_6_) δ (ppm): 2.25 (s, 3H, CH_3_), 2.64 (s, 6H, 2 × CH_3_), 7.06 (s, 2H, ArH), 7.40–7.48 (m, 1H, ArH), 7.53–7.54 (m, 1H, ArH), 7.56–7.57 (d, 1H, ArH, *J* = 6 Hz), 7.60–7.62 (m, 1H, ArH, *J* = 12 Hz), 8.73 (s, 1H, =CH), 11.81 (s, 1H, NH); ^13^C NMR (150 MHz, DMSO-*d*_6_) δ (ppm): 20.88 (CH_3_), 23.15 (2 × CH_3_), 125.46, 126.42, 127.39, 129.95, 132.12, 134.17, 136.25, 139.64 (10 × C_ar_), 142.86 (=CH), 143.98, 161.05 (2 × C_ar_).

*N*-[(2-Chloro-3-methoxyphenyl)methylidene]-2,4,6-trimethylbenzenesulfonohydrazide (**19**)

Bright yellow powder; M.p.: 158–160 °C; Yield: 47%; ^1^H NMR (600 MHz, DMSO-*d*_6_) δ (ppm): 2.25 (s, 3H, CH_3_), 2.63 (s, 6H, 2 × CH_3_), 3.85 (s, 3H, OCH_3_), 7.06 (s, 2H, ArH), 7.14–7.16 (m, 1H, ArH), 7.30–7.35 (m, 1H, ArH), 7.74–7.75 (d, 1H, ArH, *J* = 6 Hz), 8.31 (s, 1H, =CH), 11.87 (s, 1H, NH); ^13^C NMR (150 MHz, DMSO-*d*_6_) δ (ppm): 20.89 (CH_3_), 23.15 (2 × CH_3_), 56.93 (OCH_3_), 115.77, 117.87, 119.94, 123.52, 128.50, 132.15, 139.63, 141.76 (10 × C_ar_), 142.92 (=CH), 155.37, 158.99 (2 × C_ar_).

*N*-[(3-Chloro-4-methoxyphenyl)methylidene]-2,4,6-trimethylbenzenesulfonohydrazide (**20**)

Yellow powder; M.p.: 159–162 °C; Yield: 3%; ^1^H NMR (600 MHz, DMSO-*d*_6_) δ (ppm): 2.26 (s, 3H, CH_3_), 2.62 (s, 6H, 2 × CH_3_), 3.89 (s, 3H, OCH_3_), 7.05 (s, 2H, ArH), 7.29–7.30 (d, 1H, ArH, *J* = 6 Hz), 7.82–7.84 (d, 1H, ArH, *J* = 12 Hz), 7.94–7.95 (d, 1H, ArH, *J* = 6 Hz), 8.65 (s, 1H, =CH), 11.88 (s, 1H, NH); ^13^C NMR (150 MHz, DMSO-*d*_6_) δ (ppm): 20.95 (CH_3_), 24.28 (2 × CH_3_), 56.93 (OCH_3_), 111.50, 113.55, 122.14, 127.82, 129.42, 129.65, 132.99, 133.12 (10 × C_ar_), 142.15 (=CH), 157.24, 160.43 (2 × C_ar_).

*N*-[(3-Bromo-4-methoxyphenyl)methylidene]-2,4,6-trimethylbenzenesulfonohydrazide (**21**)

Pink powder; CAS Number: 1799222-06-2; M.p.: 158–160 °C; Yield: 76%; ^1^H NMR (600 MHz, DMSO-*d*_6_) δ (ppm): 2.25 (s, 3H, CH_3_), 2.64 (s, 6H, 2 × CH_3_), 3.86 (s, 3H, OCH_3_), 7.05 (s, 2H, ArH), 7.13–7.14 (d, 1H, ArH, *J* = 6 Hz), 7.49–7.50 (m, 1H, ArH), 7.71–7.72 (d, 1H, ArH, *J* = 6 Hz), 7.83 (s, 1H, =CH), 11.56 (s, 1H, NH); ^13^C NMR (150 MHz, DMSO-*d*_6_) δ (ppm): 20.88 (CH_3_), 23.18 (2 × CH_3_), 56.90 (OCH_3_), 111.50, 113.30, 127.98, 128.39, 131.00, 132.09, 133.82, 139.63 (10 × C_ar_), 142.75 (=CH), 144.14, 156.96 (2 × C_ar_).

*N*-[(5-Bromo-2-hydroxyphenyl)methylidene]-2,4,6-trimethylbenzenesulfonohydrazide (**22**)

Yellow powder; CAS Number: 1799182-63-0; M.p.: 160–163 °C; Yield: 49%; ^1^H NMR (600 MHz, DMSO-*d*_6_) δ (ppm): 2.26 (s, 3H, CH_3_), 2.62 (s, 6H, 2 × CH_3_), 6.81–6.82 (d, 1H, ArH, *J* = 6 Hz), 6.96–6.97 (d, 1H, ArH, *J* = 6 Hz), 7.07 (s, 2H, ArH), 7.51–7.55 (m, 1H, ArH), 8.11 (s, 1H, =CH), 10.39 (s, 1H, OH), 11.70 (s, 1H, NH); ^13^C NMR (150 MHz, DMSO-*d*_6_) δ (ppm): 20.89 (CH_3_), 23.07 (2 × CH_3_), 111.06, 118.96, 119.40, 128.53, 132.18, 139.59, 141.83 (9 × C_ar_), 142.94 (=CH), 155.83, 158.13, 161.21 (3 × C_ar_).

*N*-[(3,5-Dichloro-2-hydroxyphenyl)methylidene]-2,4,6-trimetylbenzenesulfonohydrazide (**23**)

Yellow powder; M.p.: 154–158 °C; Yield: 69%; ^1^H NMR (600 MHz, DMSO-*d*_6_) δ (ppm): 2.26 (s, 3H, CH_3_), 2.62 (s, 6H, 2 × CH_3_), 7.10 (s, 2H, ArH), 7.44–7.45 (d, 1H, ArH, *J* = 6 Hz), 7.75–7.76 (d, 1H, ArH, *J* = 6 Hz), 8.15 (s, 1H, =CH), 10.72 (s, 1H, OH), 12.11 (s, 1H, NH); ^13^C NMR (150 MHz, DMSO-*d*_6_) δ (ppm): 20.91 (CH_3_), 22.99 (2 × CH_3_), 120.78, 124.0, 126.69, 130.35, 132.31, 139.60, 143.25 (9 × C_ar_), 143.75 (=CH), 151.37, 153.80, 164.02 (3 × C_ar_).

*N*-[(2-Hydroxy-3,5-diiodophenyl)methylidene]-2,4,6-trimethylbenzenesulfonohydrazide (**24**)

Yellow powder; M.p.: 280 °C; Yield: 66%; ^1^H NMR (600 MHz, DMSO-*d*_6_) δ (ppm): 2.27 (s, 3H, CH_3_), 2.61 (s, 6H, 2 × CH_3_), 7.10 (s, 2H, ArH), 7.74–7.75 (d, 1H, ArH, *J* = 6 Hz), 7.99–8.00 (d, 1H, ArH, *J* = 6 Hz), 8.03 (s, 1H, =CH), 11.04 (s, 1H, OH), 12.20 (s, 1H, NH); ^13^C NMR (150 MHz, DMSO-*d*_6_) δ (ppm): 20.91 (CH_3_), 22.94 (2 × CH_3_), 83.40, 88.67, 121.45, 132.37, 132.96, 138.26, 139.58 (9 × C_ar_), 143.34 (=CH), 145.43, 146.98, 155.86 (3 × C_ar_).

*N*-[(2-Bromo-3-hydroxy-4-methoxyphenyl)methylidene]-2,4,6-trimethylbenzenesulfonohydrazide (**25**)

Brown powder; CAS Number: 1799215-12-5; M.p.: 180 °C; Yield: 55%; ^1^H NMR (600 MHz, DMSO-*d*_6_) δ (ppm): 2.25 (s, 3H, CH_3_), 2.63 (s, 6H, 2 × CH_3_), 3.83 (s, 3H, OCH_3_), 7.02–7.03 (d, 1H, ArH, *J* = 6 Hz), 7.05 (s, 2H, ArH), 7.11–7.13 (m, 1H, ArH), 8.20 (s, 1H, =CH), 8.91 (s, 1H, OH), 11.61 (s, 1H, NH); ^13^C NMR (150 MHz, DMSO-*d*_6_) δ (ppm): 20.89 (CH_3_), 23.20 (2 × CH_3_), 56.80 (OCH_3_), 111.57, 117.25, 119.60, 125.89, 132.10, 139.61, 142.76, 144.16 (10 × C_ar_), 144.92 (=CH), 149.85, 161.11 (2 × C_ar_).

2,4,6-Trimethyl-*N*-(phenylmethylidene)benzenesulfonohydrazide (**26**)

White powder; CAS Number: 16182-18-6; M.p.: 131–133 °C; Yield: 10%; ^1^H NMR (600 MHz, DMSO-*d*_6_) δ (ppm): 2.24 (s, 3H, CH_3_), 2.65 (s, 6H, 2 × CH_3_), 7.05 (s, 2H, ArH), 7.35–7.40 (m, 3H, ArH), 7.49–7.51 (m, 2H, ArH), 7.91 (s, 1H, =CH), 11.59 (s, 1H, NH); ^13^C NMR (150 MHz, DMSO-*d*_6_) δ (ppm): 20.88 (CH_3_), 23.21 (2 × CH_3_), 126.95, 129.29, 130.35, 132.10, 133.82, 134.30, 139.68, 142.76 (12 × C_ar_), 145.73 (=CH).

Examples of ^1^H NMR and ^13^C NMR spectra of synthesized 2,4,6-trimethylbenzenesulfonyl hydrazones are presented in the Supplementary Materials (Figures S1–S8).

### 2.2. Microbiology

#### *In Vitro* Antimicrobial Activity Assay

The examined compounds **2**–**26** were screened in vitro for antibacterial and antifungal activities according to the procedure described earlier by our group [35,36] with the use of the protocols of European Committee on Antimicrobial Susceptibility Testing (EUCAST) [37] and Clinical and Laboratory Standards Institute guidelines [38]. All the experiments were repeated three times, and representative data are presented. Detailed procedure for the in vitro antimicrobial activity assay is presented in the Supplementary Materials.

## 3. Results and Discussion

### 3.1. Chemistry

The 2,4,6-trimethylbenzenesulfonyl hydrazones **2**–**26** were synthesized on the basis of one-step condensation reaction of 2,4,6-timethylbenzenesufonohydrazide (**1**) with appropriate substituted benzaldehydes (Scheme 1). Among synthesized 2,4,6-trimethylbenzenesulfonyl hydrazones **2**–**26**, nine compounds had not been described earlier in scientific literature. Yield of the synthesis ranged from 3 to 78%. The highest yield was obtained for compound **8**—*N*-[(3-iodophenyl)methylidene]-2,4,6-trimethylbenzenesulfonohydrazide, whereas the lowest was for derivative **20**—*N*-[(3-chloro-4-methoxyphenyl)methylidene]-2,4,6-trimethylbenzenesulfonohydrazide. Synthesized compounds are stable solids and can be dissolved in DMSO at ambient temperature. All of synthesized compounds were identified by the analysis of ^1^H NMR and ^13^C NMR spectra.

The chemical structure of synthesized 2,4,6-trimethylbenzenesulfonyl hydrazones (**2**–**26**) was established with the use of the analysis of ^1^H NMR and ^13^C NMR spectra. Compounds **2**–**26** on the ^1^H NMR spectra possessed two characteristic singlet signals. First of them in the range of δ 7.83–8.98 ppm corresponded to proton in =CH group and confirmed the successful conduction of condensation reaction, whereas the other at δ 11.23–12.20 ppm corresponded to proton in NH group. In ^13^C NMR spectra for compounds **2**–**26**, we found peak for carbon atom of =CH group at δ 142.15–147.57 ppm. Other aliphatic and aromatic fragments of synthesized molecules in the ^1^H and ^13^C NMR spectra were found at the expected range of chemical shift.

### 3.2. Antimicrobial Activity

The antimicrobial activities of 2,4,6-timethylbenzenesufonohydrazide (**1**) and synthesized 2,4,6-trimethylbenzenesulfonyl hydrazones **2**–**26** were tested against reference Gram-positive (eight strains) and Gram-negative (six strains) bacteria. Moreover, antifungal effect towards yeasts belonging to *Candida* spp. was investigated. The results are shown in Table 1; Table 2. All tested 2,4,6-trimethylbenzenesulfonyl hydrazones **2**–**26** did not have activity against Gram-negative bacteria and fungi. The 2,4,6-timethylbenzenesufonohydrazide (**1**) showed moderate to mild antibacterial activity against Gram-negative bacterial strains (MIC = 250–1000 µg/mL) and similar antifungal activity against fungi from *Candida* spp. with MIC values in the range of 250–1000 µg/mL. Moreover, among 2,4,6-trimethylbenzenesulfonyl hydrazones, compounds **5**, **8**, **10**, **11**, **13**, **14**, **18**, and **26** were inactive towards all microorganisms from ATCC. The rest of the compounds displayed some antibacterial activity against Gram-positive bacteria. The microorganisms from *Staphylococcus* spp., *Enterococcus faecalis* ATCC 29212, *Micrococcus luteus* ATCC 10240, and *Bacillus* spp. were especially sensitive to compounds **7**, **22**, **23**, **24**, and **25**. Minimal inhibitory concentration (MIC) of these derivatives, which inhibited growth of bacteria, ranged from 7.81 to 500 µg/mL (MBC = 7.81–>1000 µg/mL).

Among them, especially compound **24**, exhibited a very strong bactericidal effect towards micrococci and bacilli (MIC = 7.81 µg/mL and MBC = 7.81–15.62 µg/mL) and strong bactericidal effect against staphylococci and enterococci (MIC = 15.62 µg/mL and MBC = 15.62–62.5 µg/mL). Similarly, compound **23** showed very strong or strong activity (MIC = 7.81–15.62 µg/mL and MBC = 15.62 µg/mL) towards *M. luteus* ATCC 10240 and *Bacillus* spp., and slightly lower (mainly moderate) against remaining bacteria.

In turn, compound **25** exhibited good activity against Gram-positive bacteria (MIC = 62.5–125 µg/mL and MBC = 125–>1000 µg/mL), except *S. aureus* ATCC 43300 and *E. faecalis* ATCC 29212 (moderate effect with MIC = 250 µg/mL and MBC = >1000 µg/mL).

Almost all bacteria (except enterococci) were also sensitive to compound **22**. Its minimal concentrations, which inhibited growth of bacteria, ranged from 31.25 µg/mL in the case of *B. subtilis* ATCC 6633 to 500 µg/mL for *S. aureus* strains (MBC = 500–>1000 µg/mL).

The other compounds showed moderate or mild activity against Gram-positive bacteria with MIC from 250 to 1000 µg/mL, or had no activity.

In relation to the antibacterial activity of reference substances, it is worth underlining that the activity of compound **24** possessed 2-hydroxy-3,5-diiodophenyl substituent against *M. luteus* ATCC 10240 (MIC = 7.81 µg/mL), which was eight times greater that the activity of nitrofurantoin (MIC = 62.5 µg/mL). Towards *B. subtilis* ATCC 6633, this compound showed two times higher (MIC = 7.81 µg/mL) activity than cefuroxime (MIC = 15.62 µg/mL) and eight times higher activity than ampicillin (MIC = 62.5 µg/mL). Activity of this hydrazone towards *B. cereus* ATCC 10876 was equal to the activity of nitrofurantoin (MIC = 7.81 µg/mL) and four times greater than the activity of cefuroxime (MIC = 31.25 µg/mL). Towards *Staphylococcus aureus* ATCC 29213 and ATCC 25923, its activity was equal to the activity of nitrofurantoin (MIC = 15.62 µg/mL).

The highest activity of molecule **23**, which was substituted with 2-hydroxy-3,5-dichlorophenyl substituent, was shown towards *M. luteus* ATCC 10240 (MIC = 7.81 µg/mL), and it was eight times higher than the activity of nitrofurantoin (MIC = 62.5 µg/mL) against this microorganism, whereas the MIC values of **23** against *B. subtilis* ATCC 6633 (MIC = 7.81) was two times lower than the MIC value of cefuroxime (MIC = 15.62 µg/mL) and eight times lower than for ampicillin (MIC = 62.5 µg/mL). It also showed two times higher activity (MIC = 15.62 µg/mL) towards *B. cereus* ATCC 10876 than cefuroxime (MIC = 31.25 µg/mL). Additionally, it is worth mentioning that this compound showed bactericidal effect against these microorganisms. In addition to this, the activity of compound **25** substituted with 2-bromo-3-hydroxy-4-methoxyphenyl substituent towards *B. subtilis* ATCC 6633 was equal to the activity of ampicillin (MIC = 62.5 µg/mL). The activity data of the most effective compounds (**23**, **24**, **25**) in comparison with nitrofurantoin against the reference Gram-positive bacterial strains is presented in graphical way in the Supplementary Materials (Figures S9 and S10).

The highest activity of compound **22**, which was substituted with 5-bromo-2-hydroxyphenyl, was displayed against *B. subtilis* ATCC 6633 (MIC = 31.25 µg/mL), and it was two times higher than the activity of reference compound ampicillin against this bacterium (MIC = 62.5 µg/mL). On the other hand, antibacterial activity of compound **7** that possessed 2-iodophenyl substituent against *B. subtilis* ATCC 6633 was equal to the activity of ampicillin (MIC = 62.5 µg/mL).

Analysis of all obtained results of antimicrobial activity screening also indicates that the conversion of 2,4,6-trimethylbenzenesulfonohydrazide **1** to 2,4,6-trimethylbenzenesulfonyl hydrazones resulted in a decrease in activity in some cases. 2,4,6-Trimethylbenzenesulfonyl hydrazone **26** formed in the reaction of 2,4,6-trimethylbenzenesulfonohydrazide **1** with benzaldehyde was inactive towards all tested bacterial strains. However, the use of aldehydes that contained in the phenyl ring a strong electron-donating substituent, a hydroxy group, and a second electron-withdrawing halogen atom (**23**–**25**) increased the activity against Gram-positive bacteria. When analyzing the activity of hydrazide **1** and benzenesulfonyl hydrazones, we saw that the free amino group promoted the activity against *Staphylococcus aureus* ATCC 25923, but the introduction of two halogens into the hydrazone’s phenyl ring caused an up to 16-fold increase in activity against this bacterial strain in the case of 2,4,6-trimethylbenzenesulfonyl hydrazone (**24**). In addition to this, the introduction of two electron-donating substituents in the phenyl ring caused a twofold increase in the activity of the compound **25** against this bacterial strain. The *Bacillus subtilis* ATCC 6633 was the most sensitive bacterial strain to tested 2,4,6-trimethylbenzenesylfonyl hydrazones. In its case, the free amino group was not conducive to activity.

## 4. Conclusions

In this research we designed, synthesized, and analyzed for potential antimicrobial activity a series of 2,4,6-trimethylbenzenesulfonyl hydrazones **2**–**26**. Our antimicrobial activity assay results indicated that some of the newly obtained compounds **2**–**26** showed particular activity against Gram-positive bacteria. The highest antibacterial effect was indicated for compounds **7**, **22**, **23**, **24**, and **25**. The bacteria from *Staphylococcus* spp., *Enterococcus faecalis* ATCC 29212, *Micrococcus luteus* ATCC 10240, and *Bacillus* spp. were especially sensitive to compound **24**. The minimal inhibitory concentration (MIC) values that inhibited growth of reference microorganisms for this hydrazone ranged from 7.81 to 15.62 µg/mL, indicating strong or very strong bactericidal effect of this molecule and its potential application as an antimicrobial agent. The antibacterial activity of the obtained compounds was connected both with the presence of sulfonyl hydrazone moiety in their molecules as well as with substitution with hydroxy and methoxy groups or chlorine and iodine atoms in the phenyl ring. The more substituents that were present in the aromatic ring of an aldehyde, which was used for the condensation reaction, the higher the observed activity of the resulting 2,4,6-trimethylbenzenesulfonyl hydrazones. The most potent compounds against tested bacterial strains will be applied as ligands for the synthesis of metal complexes.

## Figures and Tables

**Table 1:** The activity data of 2,4,6-timethylbenzenesufonohydrazide **1** and 2,4,6-trimethylbenzenesulfonyl hydrazones **2**–**13** and reference substances expressed as MIC (MBC) [µg/mL] and {MBC/MIC} ratio against the reference strains of Gram-positive bacteria.

Species		MIC (MBC) [µg/mL] and {MBC/MIC} Ratio of the Studied Compounds and Positive Controls											
		1	2	3	4	6	7	9	12	CIP/VA*	NIT	CFX	APC
Gram-positive bacteria	*Staphylococcus* *aureus* ATCC 25923	250 (500) {2}	-	500 (>1000) {>2}	1000 (>1000) {>1}	1000 (>1000) {>1}	1000 (>1000) {>1}	1000 (>1000) {>1}	-	0.48 (0.48) {1}	15.62 (15.62)	0.49	nd
	*Staphylococcus* *aureus* ATCC 43300	500 (500) {1}	-	1000 (>1000) {>1}	-	-	1000 (>1000) {>1}	1000 (>1000) {>1}	-	0.24 (0.24) {1}	7.81 (15.62)	nd	nd
	*Staphylococcus* *aureus* ATCC 29213	500 (500) {1}	-	-	-	-	1000 (>1000) {>1}	1000 (>1000) {>1}	-	0.48 (0.48) {1}	15.62 (15.62)	nd	nd
	*Staphylococcus* *epidermidis* ATCC 12228	500 (500) {1}	-	500 (>1000) {>2}	500 (>1000) {>2}	1000 (>1000) {>1}	1000 (>1000) {>1}	1000 (>1000) {>1}	-	0.12 (0.12) {1}	3.91 (7.81)	0.24	nd
	*Enterococcus* *faecalis* ATCC 29212	1000 (>1000) {>1}	-	500 (>1000) {>2}	-	-	-	-	-	0.98 * (1.95) {2}	7.81 (7.81)	nd	nd
	*Micrococcus* *luteus* ATCC 10240	500 (500) {1}	-	-	-	1000 (>1000) {>1}	250 (>1000) {>4}	-	250 (>1000) {>4}	0.98 (1.95) {2}	62.5 (62.5)	0.98	nd
	*Bacillus* *subtilis* ATCC 6633	500 (500) {1}	500 (>1000) {>2}	500 (>1000) {>2}	250 (1000) {>4}	125 (>1000) {>8}	62.5 (>1000) {>16}	250 (>1000) {>4}	250 (1000) {4}	0.03 (0.03) {1}	3.91 (3.91)	15.62	62.5
	*Bacillus* *cereus* ATCC 10876	500 (1000) {2}	250 (>1000) {>4}	1000 (>1000) {>1}	-	1000 (>1000) {>1}	-	-	-	0.06 (0.12) {2}	7.81 (15.62)	31.25	nd

“-“, no activity; nd, not determined. The antibacterial compounds used as positive controls: ciprofloxacin (CIP), vancomycin (VA*), nitrofurantoin (NIT), cefuroxime (CFX), and ampicillin (APC). Compounds with bactericidal effect (MBC/MIC ≤4) are marked with bolded font.

**Table 2:** The activity data of 2,4,6-trimethylbenzenesulfonyl hydrazones **14–26** and reference substances expressed as MIC (MBC) [µg/mL] and {MBC/MIC} ratio against the reference strains of Gram-positive bacteria.

Species		MIC (MBC) [µg/mL] and {MBC/MIC} Ratio of the Studied Compounds and Positive Controls													
		15	16	17	19	20	21	22	23	24	25	CIP/VA*	NIT	CFX	APC
Gram-positive bacteria	*Staphylococcus* *aureus* ATCC 25923	-	-	-	-	-	-	500 (>1000) {>2}	500 (>1000) {>2}	15.62 (15.62) {1}	125 (>1000) {>8}	0.48 (0.48) {1}	15.62 (15.62)	0.49	nd
	*Staphylococcus* *aureus* ATCC 43300	-	-	-	-	-	-	500 (>1000) {>2}	500 (>1000) {>2}	15.62 (15.62) {1}	250 (>1000) {>4}	0.24 (0.24) {1}	7.81 (15.62)	nd	nd
	*Staphylococcus* *aureus* ATCC 29213	-	500 (>1000) {>2}	-	-	-	-	500 (>1000) {>2}	250 (>1000) {>4}	15.62 (62.5) {4}	125 (>1000) {>8}	0.48 (0.48) {1}	15.62 (15.62)	nd	nd
	*Staphylococcus* *epidermidis* ATCC 12228	-	250 (>1000) {>4}	1000 (>1000) {>1}	500 (>1000) {>2}	-	-	125 (500) {4}	250 (>1000) {>4}	15.62 (15.62) {1}	125 (1000) {8}	0.12 (0.12) {1}	3.91 (7.81)	0.24	nd
	*Enterococcus* *faecalis* ATCC 29212	-	-	-	-	-	-	-	125 (>1000) {>8}	15.62 (62.5) {4}	250 (>1000) {>4}	0.98 * (1.95) {2}	7.81 (7.81)	nd	nd
	*Micrococcus* *luteus* ATCC 10240	-	1000 (>1000) {>1}	500 (>1000) {>2}	1000 (>1000) {>1}	1000 (>1000) {>1}	1000 (>1000) {>1}	1000 (>1000) {>1}	7.81 (15.62) {2}	7.81 (15.62) {2}	125 (250) {2}	0.98 (1.95) {2}	62.5 (62.5)	0.98	nd
	*Bacillus* *subtilis* ATCC 6633	1000 (>1000) {>1}	125 (1000) {8}	250 (1000) {4}	250 (1000) {4}	1000 (>1000) {>1}	500 (>1000) {>2}	31.25 (500) {16}	7.81 (15.62) {2}	7.81 (7.81) {1}	62.5 (125) {2}	0.03 (0.03) {1}	3.91 (3.91)	15.62	62.5
	*Bacillus* *cereus* ATCC 10876	1000 (>1000) {>1}	1000 (>1000) {>1}	-	-	-	500 (>1000) {>2}	250 (1000) {4}	15.62 (15.62) {1}	7.81 (15.62) {2}	125 (>1000) {>8}	0.06 (0.12) {2}	7.81 (15.62)	31.25	nd

“-“, no activity; nd, not determined. The antibacterial compounds used as positive controls: ciprofloxacin (CIP), vancomycin (VA*), nitrofurantoin (NIT), cefuroxime (CFX), and ampicillin (APC). Compounds with bactericidal effect (MBC/MIC ≤4) are marked with bolded font.

## Data Availability

Data are contained within the article or supplementary material.

## Supplementary Materials

The following are available online at https://www.mdpi.com/article/10.3390/ma14112723/s1. Figure S1. ^1^H NMR spectrum of compound **7**—*N*-[(2-iodophenyl)methylidene]-2,4,6-trimethylbenzenesulfonohydrazide. Figure S2. ^13^C NMR spectrum of compound **7**—*N*-[(2-iodophenyl)methylidene]-2,4,6-trimethylbenzenesulfonohydrazide. Figure S3. ^1^H NMR spectrum of compound **15**—*N*-[(4-bromophenyl)methylidene]-2,4,6-trimethylbenzenesulfonohydrazide. Figure S4. ^13^C NMR spectrum of compound **15**—*N*-[(4-bromophenyl)methylidene]-2,4,6-trimethylbenzenesulfonohydrazide. Figure S5. ^1^H NMR spectrum of compound **21**—*N*-[(3-bromo-4-methoxyphenyl)methylidene]-2,4,6-trimethylbenzenesulfonohydrazide. Figure S6. ^13^C NMR spectrum of compound **21**—*N*-[(3-bromo-4-methoxyphenyl)methylidene]-2,4,6-trimethylbenzenesulfonohydrazide. Figure S7. ^1^H NMR spectrum of compound **26**—2,4,6-trimethyl-*N*-(phenylmethylidene)benzenesulfonohydrazide. Figure S8.^13^C NMR spectrum of compound **26**—2,4,6-trimethyl-*N*-(phenylmethylidene)benzenesulfonohydrazide. Figure S9. The activity data of the most active compounds: (a) **23**, (b) **24**, (c) **25**, and (d) nitrofurantoin against the reference Gram-positive bacterial strains used in antimicrobial activity study. Figure S10. The activity data of the most active compounds (**23**, **24**, and **24**) compared to nitrofurantoin and expressed as MIC (µg/mL) against the reference Gram-positive bacterial strains.
